# Improving safety and efficacy with pharmacist medication reconciliation in orthopedic joint surgery within an enhanced recovery after surgery program

**DOI:** 10.1186/s12913-022-07884-9

**Published:** 2022-04-06

**Authors:** Xiaoying Zheng, Lei Xiao, Ying Li, Feng Qiu, Wei Huang, Xinyu Li

**Affiliations:** 1 Department of Pharmacy, The First Affiliated Hospital of Chongqing Medical University, Yuzhong District, 1 Youyi Rd, Chongqing, China; 2 Department of Pharmacy, Chongqing Health Center for Women and Children, Chongqing, China; 3 Department of Orthopedic Surgery, The First Affiliated Hospital of Chongqing Medical University, Chongqing, China

**Keywords:** Medication reconciliation, Medication discrepancy, Enhanced recovery after surgery, Periprosthetic joint infection

## Abstract

**Purpose:**

To investigate the impact of medication reconciliation (MR), through avoidance of unintentional medication discrepancies, on enhanced recovery after surgery programs designed for older patients undergoing orthopedic joint surgery.

**Method:**

Our study was divided into two phases. In the first phase, MR was performed for elderly patients undergoing orthopedic joint surgery. Types of medication discrepancies and their potential risks were analyzed. In the second phase, a controlled study was conducted in a subgroup of patients diagnosed with periprosthetic joint infection (PJI) and who were scheduled for two-stage revision. The primary goal was to investigate the impact of MR on length of stay for the first stage. The secondary goal was to investigate the time between the first admission and the reimplantation of a new prosthesis, the number of readmissions within 30 days, hospitalization cost.

**Results:**

A total of 506 medication discrepancies were identified in the included 260 patients. Intolerance had the highest incidence (*n* = 131, 25.7%). The Bayliff tool showed that 71.9% were assessed as level 2 risk, and 10.3% had a life-threatening risk. For patients with PJI, MR reduced the average length of stay in the first stage (16.3 days vs. 20.7 days, *P* = 0.03) and shortened the time (57.3 days vs. 70.5 days, *P* = 0.002) between the first admission and the reimplantation of a new prosthesis. The average cost of hospital stay ($8589.6 vs. $10,422.6, *P* = 0.021), antibiotics ($1052.2 vs. $1484.7, *P* = 0.032) and other medications ($691.5 vs. $1237.6, *P* = 0.014) per patient at our hospital were significantly decreased. Notably, significant improvements in patient satisfaction were seen in participants in the MR group.

**Conclusion:**

Through MR by clinical pharmacists, medication discrepancies within the orthopedic ERAS program could be identified. For patients with periprosthetic joint infection, better patient satisfaction and clinical and economical outcomes can be achieved with this method.

## Introduction

Enhanced recovery after surgery (ERAS) protocols have gained broad acceptance in many surgical disciplines [1]. To reduce patients’ surgical stress response, optimize their physiologic function and facilitate recovery, the integrated continuum constitutes the implementation of over 20 evidence-based strategies, many of which employ pharmacologic therapies [2]. On the other hand, ERAS is supported by multidisciplinary participation and the integration of regimens from a variety of specialized areas. Transitions of hospital stay and changes in patient conditions before and after surgery require more detailed modification of the pharmacological regimen [3, 4]. As such, risk assessment addressing pharmacotherapy-related decisions during the transition of care and in each perioperative phase is essential for compliance with ERAS pathways and medication error prevention. 

Medication reconciliation (MR) is a formal process or technique used by health care providers and pharmacists to gather a complete and accurate list of a patient's prescribed and home medications. By comparing the items on the list with the current drug regimen, discrepancies were identified in different levels of care, care settings, or points in time. This information was used to inform prescribing decisions and to identify and prevent medication discrepancies [5, 6]. It is widely recommended to avoid unintentional medication discrepancies between patients’ medications across transitions in care. The implementation of ERAS protocols is also challenged by the potential medication discrepancies as discussed above, and MR may provide an opportunity for addressing this issue.

In recent years, there has been a rapid adoption of ERAS pathways in many Chinese hospitals [7–13], and clinical pharmacists have been actively involved in ERAS program implementation. In the First Affiliated Hospital of Chongqing Medical University, clinical pharmacists have been trying to incorporate MR into the framework of ERAS for orthopedic surgery in older patients (≥ 65 years). With complicated preoperative clinical conditions, preexisting functional limitations and polypharmacy in ERAS programs, older patients carry an even higher risk for perioperative medication discrepancies. Some studies suggested that older patients showed a consistent correlation with the prevalence of clinically significant unintentional discrepancies [14, 15]. Thus, it is necessary for hospitals to perform a complete and accurate MR for older patients in orthopedic joint surgery. Many studies [16, 17] have indicated that MR for improving the appropriateness of medications in hospitalized older orthopedic patients may be associated with better patient outcomes compared with other settings. However, these patient outcomes have often been limited to readmission, emergency department visits and the occurrence of all-cause death, a new fracture, myocardial infarction and ischemic stroke. Furthermore, few trials have evaluated the effect of MR on the length of stay, medical cost, and patient satisfaction for older patients undergoing orthopedic joint surgery.

The purpose of this study was to investigate the types of medication discrepancies and their potential risks for older patients undergoing orthopedic joint surgery in the first phase. For the second phase, the controlled study of PJI patients scheduled for two-stage revision, the primary goal was to investigate the impact of MR on length of stay for the first stage. The secondary goal was to investigate the time between the first admission and the reimplantation of a new prosthesis, readmission within 30 days, hospitalization cost.

## Methods

### Study Setting

This study was performed from September 2019 to December 2020 in the joint surgery ward of a large tertiary care academic hospital in Chongqing, China. All methods were carried out in accordance with relevant guidelines and regulations. This study was approved by the institutional ethics board of The First Affiliated Hospital of Chongqing Medical University (Chongqing, China) in 2020. Informed consent was obtained from all patients by signing the paper version of agreement.

## The first-phase study

### Protocol and data collection

The study was divided into two phases. The first phase was planned as a preliminary study for analysis of discrepancies and their potential risks. Reconciliation was provided to all included patients, and no control study was made.

### Inclusion and exclusion criteria

In the first phase, all patients who underwent elective orthopedic joint surgery were included in the ERAS pathway. They were then screened for the following criteria: (1) age ≥ 65 years; (2) prescribed with at least 1 medication after admission. Patients who met the above criteria were included in the study. Patients were excluded if they met any of the following criteria: (1) patients whose medication history was not accessible or unreliable for any reason; (2) patients with language or hearing or mental disorder who could not communicate with the pharmacists; (3) patients who were not present in the room during pharmacists’ visits within 24 h following admission; and (4) hospitalization time less than 72 h.

### Medication reconciliation

To implement MR successfully, roles and workflows based on interprofessional collaboration of orthopedic surgeons, nurses, and clinical pharmacists were constructed. Medication orders of patients were reviewed by clinical pharmacists within 24 h of admission. First, the documented medication history and admitting medication orders (medication, dose, route, frequency) were collected. Newly updated laboratory tests were also reviewed. After reviewing the present medical orders, a clinical pharmacist interviewed each patient (or their family caregivers) to obtain their medication history. This information was then compared with admitting medication orders. Discrepancies were defined as differences between present medication orders based on documented medication history and direct interview and assessment.

Second, based on the consented pharmacotherapeutic options within each ERAS element for joint surgery (developed by the surgical team and pharmacists, Table 1), the pharmacist also checked the pre- and postoperative medication orders for discrepancies from the ERAS options.

**Table 1:** The consented pharmacotherapeutic options within each ERAS element for orthopedic joint surgery

Drugs	Pre-surgery	Post-surgery
Antihypertensive drugs	Stop reserpine at least 5 days before the surgery	Continue with previous antihypertensive regimen, reserpine be substituted with other antihyperten- sives (calcium channel blockers, angiotensin- converting enzyme inhibitors, et al.)
	Avoid acute withdrawal of a beta blocker	
	Withhold angiotensin converting enzyme inhibitors and angiotensin receptor blockers on the morning of surgery. For heart failure or poorly controlled hypertension, continue to avoid further exacerbation of these conditions	
Antidiabetic drugs	Not achieving goals: switch sulfonylurea to insulin glargine, insulin detemir or for other basic insulin; insulin lysine before meals	Continue with insulin therapy if necessary, for a stable glycemic control
	Consider adding metformin according to the blood glucose level	
Preoperative analgesia	Pain assessment	Pain assessment
	Adding NSAIDs, selective cyclooxygenase-2 inhibitors preferred if numeric rating scales for pain > 3	Parecoxib or flurbiprofen(i.v) for 3 days, then continue with celecoxib (P.O.)if necessary
	Adding pregabalin or duloxetine (venlafaxine) for neuropathic pain	Adding pregabalin or duloxetine (venlafaxine) for neuropathic pain
	Adding tramadol (P.O) or acetaminophen if necessary	Adding tramadol (P.O) or acetaminophen if necessary
	Screening for mistakenly combination of two NSAIDs, tramadol (PCIA) and tramadol (P.O.), tramadol and duloxetine (venlafaxine)	Screening for mistakenly combination of two NSAIDs, tramadol (PCIA) and tramadol (P.O.), tramadol and duloxetine(venlafaxine)
	Parecoxib 40 mg (iv) before induction	
Corticosteroids (patients who are now using or have history of using corticosteroids	Evaluation of HPA axis suppression	
	Continue with current corticosteroids therapy	Continue with current cor- ticosteroids therapy
	For suppressed the HPA axis: hydrocortisone infusion 100 mg before anesthetic induction,50 mg q8h for 24 h after sugery → 25 mg q8h, 24 h → 50 mg qd, 24 h → discontinue	For suppressed HPA axis: hydrocortisone infusion 50 mg q8h for 24 h after sugery → 25 mg q8h, 24 h → 50 mg qd,24 h → discontinue (evaluation of symptoms like nausea /vomiting/ tachycardia/ hyponatremia / hypotension)
Medication affecting hemostasis	Discontinue aspirin or clopidogrel at least 5 days before the surgery, switching to low molecular weight heparin (LMWH) if necessary	Resumption of original antithrombotic therapy 24 h after surgery, typically the evening of the day of surgery or the evening of the day after surgery, as long as adequate hemostasis has been achieved
	Discontinue rivaroxaban, dabiga-tran, apixaban for 3 days before the surgery, switching toLMWH if necessary	
	Discontinue warfarin after admission, bridging to LMWH	
	Discontinue LMWH 12 or 24 h before the surgery	
Medicine for sleep disorder	Patients with new developed insomnia: screening and evaluating of medication that may disturb sleep (theophylline, steroids et al.) adjust timing of administration to avoid disturbance at night	The same strategy as before surgery
	Patients with new insomnia (Nonpharmacologic strategies not effective) initiation of non-benzodiazepines: zolpidem/ zopiclone	
	For patients with anxiety or reduced total sleep time: benzodi-azepines (estazolam, apozolam). Long-acting benzodiazepines (clonazepam) should be avoided in older adults	
Antipsychotics for delirium	Assessment of delirium especially for senior patients with Alzheimer disease	The same strategy as before surgery
	Assessment of pain	
	Initiation of small dose quetiapine, olanzapine if delirium presented	
	Low-dose haloperidol (0.5 to 1 mg) be used as needed to control moderate to severe agitation (avoided in patients with parkinsonism)	
Prophylactic antibiotics	Cefuroxime or cefazolin infusion 30 min before incision	Antibiotic prevention order should be discontinued within 24 h after surgery
	Vancomycin infusion 1–2 h before incision	
	Clindamycin infusion 30 min before incision if patients are allergic to Cephalosporins	
Antibiotic treatment for PJI	Microbial cultivation (synovial fluid or blood) before initiation of antimicrobial therapy	Before transferring to other hospital: verification of medication supply in accordance with the present regimen
	Empiric therapy: vancomycin combined with levofloxacin/ a third- or fourth-generation cephalosporin/ piperacillin- tazobactam	
	Definitive therapy should be based on the culture results and the effect of antibiotics used	
	For patients with S. aureus PJI and residual hardware following surgery (eg, patients who undergo debridement with retention or patients who undergo one-stage exchange), using rifampin in combination with at least one other anti-staphylococcal agent	
	Vancomycin level monitoring and dosage/interval adjustment to reach the trough level of 15–20 mg L^−1^	

Third, once the patient’s condition was stabilized after surgery, he was discharged or transferred to nonacute care facilities (the country’s medical transfer policy encourages medical transference from higher-grade hospitals to lower-grade hospitals once the patient’s status was stabilized but follow-up treatment is still required). Within 24 h before discharge or transfer, discharge orders were checked to ensure the continuance of the pharmacotherapy regimen if needed (e.g., pain medications, anticoagulants, antibiotics). If medications on the discharge list were not available in the transferred hospitals (information obtained by verification with counterpart pharmacists through phone calling), discrepancies were also identified.

After verification by the ordering surgeon, discrepancies that occurred in response to a patient’s change in his clinical status or due to formulary substitutions were considered intentional discrepancies (ID). Those unjustified discrepancies were classified as an unintentional discrepancy (UD).

For the unintentional discrepancies, once they were identified, pharmacists made the following changes: discontinue medication, add medication, continue at different doses/frequencies/routes/manufacturers of medication, or substitute with a different medication. These changes were documented, and a new reconciliated medication list was formed. Then, surgeons were notified for clarification and shared with the reconciliated medication list (pharmacotherapy regimen). Discussion in the surgical team was sometimes held for complicated cases. During the entire process, nurses responsible for admitting procedures and for administering drugs would notify the pharmacists for reconciliation; new medication lists were also shared after surgeon verification.

Finally, pharmacists confirmed the reconciliation by reviewing the current medical order again, and patient education was provided if necessary.

### Analysis of medications implicated in discrepancies

Medications related to medication discrepancy in our study were classified into twelve different types: cardiovascular agents, analgesics, antimicrobials, antipsychotics, antithrombotics, hypnotics and antipsychotics, insulin and oral hypoglycemic agents, glucocorticoids, antiemetics and laxatives, immunomodulatory drugs, antiemetics and gastrointestinal agents, nutritional agents and others.

### Classification of discrepancies

Discrepancies were classified into eight different types: omission, commission (mistaken addition of a medication), intolerance (either because of the patients’ specific conditions like allergy, or because it carried risks for a surgical procedure), different dose or route or frequency, different medication, and continuation of orders that needed to be stopped, duplication, interaction, and those uncategorized.

### Assessment of potential hazards of discrepancies

At the end of this study, the potential hazard of each UD was assessed by a surgeon and a clinical pharmacist using the Bayliff tool [18], which categorizes the severity of hazard into four levels. Level 0- No clinical impact, Level 1-Potential, mild clinical impact, Level 2-Potential clinical impact leading to further treatment or lengthened hospital stay, Level 3- Life-threatening. Assessment occurred independently between the surgeon and the pharmacist. If they assessed the severity of hazard differently, then the more severe hazard was recorded as the result of assessment.

## The second phase

### Study protocol and data collection

**Fig.1:**
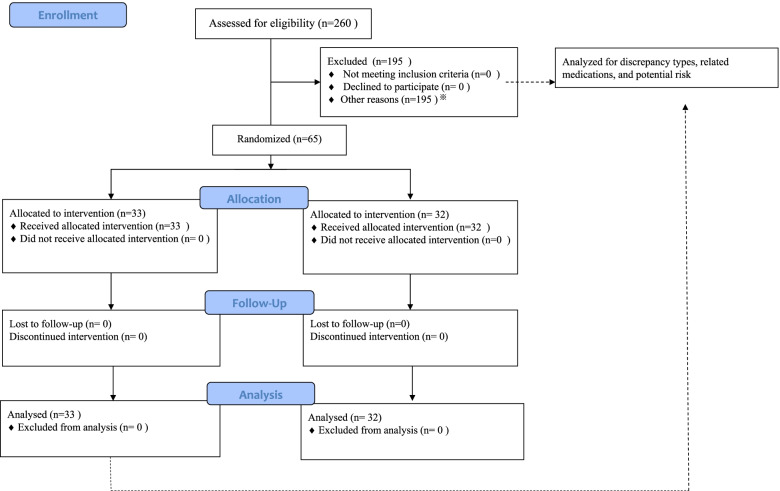
Flowchart showing enrollment of patients

In the second phase, to study the impact of MR, a controlled study was conducted between the intervention group (with pharmacist-led reconciliation) and the control group (without reconciliation). Patients who met the inclusion criteria of the first phase and were diagnosed with PJI were asked for consent prior to randomization. When consent was obtained, individuals were then randomly assigned to either of two groups. Randomization online software was used to generate a randomization plan (http://www.randomization.com), which assigned patients into intervention (1) and control (0) groups (Fig. 1). All patients received two-stage revision for the treatment of PJI. This treatment consists of removing the prosthesis and cement in the infected area and inserting an antibiotic-impregnated spacer (stage 1 or first stage), usually made from bone cement. When infection has cleared or controlled with administration of antibiotics, a revision total joint is implanted (stage 2 or second stage). Surgery in both stages was performed in our surgery ward.

### Outcomes

The primary outcome was length of stay for the first stage. Secondary outcomes were length of stay for the second stage, readmission within 30 days, unplanned outpatient visits between the two stages and within 3 months following the second stage, the time between the first admission and the reimplantation of a new prosthesis, hospitalization cost of PJI per patient in our hospital and the post-acute care facility, and patient satisfaction.

### Power analysis

The sample size calculation was based on the primary outcome: length of stay for the first stage. Using data from the preliminary studies (length of stay for the first stage: 22.3 ± 4.1 days in the control group; 17.8 ± 5.2 days for the reconciliation group) and a power of 90% as well as an alpha level of 5%, the study required 24 patients in each arm. To account for dropouts and losses to follow-up, the number of patients was increased by 30%. Consequently, 33 patients were recruited in each arm.

### Statistical analyses

Statistical analysis was performed using the SPSS version 17 statistical package (SPSS, Chicago, IL). The differences in the primary endpoint between the intervention group and the control group were evaluated using Fisher’s exact test. Descriptive statistics consisted of the mean and standard deviation (SD). A value of *P* < 0.05 was considered statistically significant.

## Results

### The first-phase study

In the first phase, from September 2019 to March 2020, 305 patients were screened, and 260 were included in the study. Data from these patients were analyzed for types of discrepancies, categories of related medications and potential hazards. A total of 4503 medication orders were screened, and 506 unjustified medication discrepancies were detected and reconciled involving 260 patients, with a mean of 1.9 per patient (SD = 0.6). The average age of patients with medication order discrepancies was 68.4 years, among whom 65.9% were women. The other baseline demographic and clinical characteristics are shown in Table 2.

**Table 2:** Baseline demographic and clinical characteristics

Gender	N(260)	%
Male	89	34.3
Female	171	65.9
Age	68.4 ± 13.2	
Diagnosis	N	%
Knee osteoarthritis	65	25.0
Femoral neck fracture	55	21.2
Prosthetic joint infection	23	8.84
Intertrochanteric fracture	30	11.5
Rheumatoid arthritis	13	5.0
Avascular necrosis of femoral head	10	3.8
Developmental Dysplasia of the Hip	10	3.8
Hip osteoarthritis	10	3.8
Shoulder sleeve injury	7	2.7
Bone tuberculosis	7	2.7
Meniscus injury	5	1.9
Cruciate ligament injury	5	1.9
Recurrent patellofemoral dislocation	3	1.2
Subacromial impingement syndrome	3	1.2
Shoulder dislocation	2	0.8
Hemophilic arthritis	2	0.8
Talus necrosis	2	0.8
Tibial plateau fracture	2	0.8
Ankle osteoarthritis	2	0.8
Osteomyelitis	2	0.8
Radial neck fracture	2	0.8

### Medications implicated in medication discrepancies

Among the medications most frequently pertaining to discrepancies, cardiovascular agents had the highest incidence (22.5%, *n* = 114), followed by analgesics (17.0%, *n* = 86) and antibacterial drugs (13.0%, *n* = 66) (Table 3).

**Table 3:** Medications implicated in medication discrepancy

Medication	N	%
Cardiovascular agent	114	22.5
Analgesics	86	17.0
Antimicrobials	66	13.0
Antithrombotic	58	11.5
Hypnotics and antipsychotics	56	11.1
Insulin and oral hypoglycemic agents	36	7.1
Glucocorticoids	28	5.5
Anti-emetics and laxatives	18	3.6
Immunomodulatory drugs	18	3.6
Antiemetics and Gastrointestinal Agent	14	2.8
Nutritional	6	1.2
Other	6	1.2
Total	506	100.0

### Types of discrepancies

According to the categorization, the occurrence of medication discrepancies was listed as intolerance (*n* = 131, 25.7%), which was the highest rate, followed by omission (*n* = 112, 22.1%), prolonged duration of therapy (*n* = 70, 13.8%), discrepancy in drug dose, route, or frequency (*n* = 66, 13.0%), duplication (*n* = 61, 12.1%), commission (*n* = 35, 6.9%), interaction (*n* = 25, 4.9%), and uncategorized (*n* = 8, 1.6%). Typical examples of discrepancies are illustrated in Table 4.

**Table 4:** Types and examples of discrepancies

Types of Discrepancies	**N**	**%**	examples of discrepancies
Intolerance	131	25.7	NSAIDs (ibuprofen, celecoxib, et al.) prescribed for patients with severe renal impairment; aspirin (or clopidogrel) prescribed before surgery; continued use of antihypertensives should not be ordered (reserpine) before surgery; anticoagulants prescribed for patients with extensive ecchymosis after surgery
Omission	112	22.1	Metformin for type II diabetic patients; sedatives for patients with delirium after surgery; medication for hypertension after surgery; prophylactic antibiotics before surgery; mono-drug therapy for uncontrolled pain; omission of antifungals for infection by fungus; omission of perioperative glucocorticoids in patients with adrenocortical hypofunction (caused by long term irrational use of glucocorticoids)
Prolonged duration of therapy	70	13.8	Prolonged use of prophylactic antibiotics /Analgesics/Anti-Emetics/iron saccharate
Discrepancy in drug dose/ route/ frequency	66	13.0	Continued use of current vancomycin dose/frequency with trough drug concentration outside therapeutic range; once daily LMWH for patients with deep vein thrombosis; once/twice daily cephalosporins or piperacillin tazobactam; levofloxacin 200 mg once daily
Drug duplication	61	12.1	Rivaroxaban, aspirin and low molecular heparin were used as a combination for patients with deep vein thrombosis and coronary heart disease; Flurbiprofen(Patient controlled analgesia)and parecoxib(IV push)were prescribed after surgery
Commission	35	6.9	Moxifloxacin prescribed for urinary tract infection; Cefuroxime prescribed for infection of methicillin-resistant Staphylococcus aureus
Drug Interaction	25	4.9	Rivaroxaban prescribed with voriconazole(or rifampin)
Uncategorized	8	1.6	Patients had been using painkillers bought from Thailand which contained 5 mg of dexamethasone per pill. As pharmacist obtained this information from patients, serum cortisol level was checked upon pharmacist' advice and adrenocortical hypofunction was later diagnosed with this patient. Intravenous corticosteroid was then prescribed perioperatively
Total	506	100	

### Potential hazards of discrepancies

For the assessment of potential hazards of discrepancies, the Bayliff tool showed that all medication discrepancies carried the potential hazard. A total of 364 (71.9%) patients were classified as level 2 (with potential clinical impact leading to further treatment or lengthened hospital stay), 90 (17.8%) as level 1 (mild potential clinical impact), and 52 (10.3%) as a life-threatening risk (level 3) (Table 5). Discrepancies in the severity of hazard assessments occurred in 3 cases (0.6%).

**Table 5:** Potential risk assessment of medication discrepancies by Bayliff tool

Risk classification	N	%
Level 0: No clinical impact	0	0
Level 1: mild potential clinical impact	90	17.8
Level 2: potential clinical impact leading to further treatment or lengthened hospital stay	364	71.9
Level 3: Life-threatening	52	10.3

### The second-phase study

To study the impact on hospital expenses and patient satisfaction in the second phase of this study from April 2020 to December 2020, 69 PJI patients were screened, and 65 patients were included. Using a computer-generated randomized table, these patients were randomly allocated to the intervention group (*n* = 33) and control group (without MR, *n* = 32).

### The effect of MR on hospital utilization

There was no significant difference in the baseline characteristics of PJI patients between the intervention and control groups (Table 6). The intervention reduced the average length of stay for the first stage for the intervention group (16.3 days vs. 20.7 days, *P* = 0.03). There were no readmissions or unplanned outpatient visits within 30 days of discharge in the intervention group compared with 3 admissions and 4 unplanned visits in the control group, although no statistical significance was reached. Notably, the time between the first admission and the reimplantation of a new prosthesis in the intervention group was significantly shortened (57.3 days vs. 70.5 days, *P* = 0.002) (Table 6).

**Table 6:** the effect of medication reconciliation on hospital utilization of patients with PJI

Hospital utilization	Reconciliation *n* = 33	Control *n* = 32	P
Age (mean)	67.4 ± 4.5	68.2 ± 5.8	0.633
BMI (mean)	25.3 ± 3.7	25.7 ± 2.1	0.311
Male, n (%)	13 (53.5)	16 (50.0)	0.167
Female, n (%)	20 (46.5)	16 (50.0)	0.154
Length of stay for the first stage (days)	16.3 ± 3.8	20.7 ± 3.4	0.03
Length of stay for the second stage (days)	9.6 ± 2.7	10.2 ± 3.2	0.12
Readmission within 30 Days, n (%)	0 (0.0)	2 (6.25)	0.33
Unplanned outpatient visits between the two stages and within 3 months following the second stage, n (%)	0 (0.0)	4 (12.5)	0.09
The time between the first admission and the reimplantation of a new prosthesis (days)	57.3 ± 7.2	70.5 ± 11.9	0.002

### Economic impact of MR

The economic impact of MR is shown in Table 7. With MR, the average cost of hospital stay at our hospital per patient was decreased ($8589.6 vs. $10,422.6, *P* = 0.021). Declination was also at the cost of medication ($1052.2 vs. $1484.7, *P* = 0.032) and antibiotics ($691.5 vs. $1237.6, *P* = 0.014). The post-acute care facility also saw the same trend in cost decline, $3229.3 vs. $4194.1 for total medical cost (*P* = 0.056); however, changes in medication ($1241.3 vs. $1305.3, *P* = 0.912) and antimicrobials cost ($981.7 vs. $1153.7, *p* = 0.462) did not reach significance.

**Table 7:** The effect of medication reconciliation on hospitalization cost of PJI per patient in our hospital and the post-acute-care facility

cost	our hospital (USD)			the post-acute-care facility (USD)		
	reconciliation	no reconciliation	P	reconciliation	no reconciliation	P
Total	8589.6 ± 1002.1	10,422.6 ± 1173.3	0.021	3229.3 ± 490.2	4194.1 ± 895.0	0.056
Medication	1052.2 ± 256.3	1484.7 ± 328.1	0.032	1241.3 ± 278.1	1305.3 ± 331.4	0.912
Antimicrobial	691.5 ± 241.8	1237.6 ± 300.2	0.014	981.7 ± 215.4	1153.7 ± 104.5	0.462

### The effect of MR on patient satisfaction

All enrolled PJI patients in the intervention group and control group completed the survey and provided rating scores about patient satisfaction on the day of discharge or transfer. The survey constituted 10 questions encompassing critical aspects of the surgical experience (Table 8). A 10-point scale for self-report assessment was used for each question.

**Table 8:** The survey of patient’s satisfaction

question	reconciliation (means ± SD)	Control (means ± SD)	p
1. Health information materials were effective	8.9 ± 3.3	8.5 ± 4.3	0.071
2. The operating room staff were caring and attentive to my needs	9.3 ± 2.1	7.5 ± 2.6	0.092
3.After my surgery, pain was kept at a level that was acceptable to me	8.4 ± 1.8*	6.2 ± 1.8	0.035
4.After my surgery, if I experienced nausea or vomiting, it was kept to a level that was acceptable to me	8.9 ± 2.1*	6.7 ± 4.6	0.043
5.After my surgery, I was able to get my questions answered adequately by members of the healthcare team	7.6 ± 2.5	7.4 ± 3.2	0.931
6.The surgical unit staff were caring and attentive to my needs	8.8 ± 2.1	8.1 ± 4.2	0.056
7.I received enough information to care for myself and felt ready to go home when I was discharged	9.7 ± 1.8*	7.7 ± 1.6	0.042
8.After discharge, I knew whom to contact if I had a question or concern	7.9 ± 3.7	7.8 ± 2.4	0.326
9.My surgical experience matched what I understood it would be	7.4 ± 1.2	7.1 ± 1.8	0.671
10. I was satisfied with the quality of the care I received	8.6 ± 2.4	7.2 ± 1.6	0.608

^*^p < 0.05 compared with control

We observed an increase in rating scores in all the aspects surveyed in Table 8. Notably, significant improvements were seen in three aspects: perioperative pain management (8.4 ± 1.8 points compared with 6.2 ± 1.8 points), management of nausea and vomiting (8.9 ± 2.1 points compared with 6.7 ± 4.6 points), enough information received and feeling of readiness at discharge (9.7 ± 1.8 points compared with 7.7 ± 1.6 points).

## Discussion

Through the MR led by pharmacist, unjustified medication discrepancies were effectively identified, which not only prevented unintentional medication discrepancies, but also improved patients satisfaction, achieved a better surgical outcome in PJI patients as well as reduced medical cost. To the best of our knowledge, this is the first study adding the work of reconciliation to the medication management for ERAS in older patients undergoing orthopedic joint surgery. Through MR in the first stage, medication discrepancies were effectively recognized. Among the medications implicated in medication discrepancies, cardiovascular agents were found to have the highest incidence. Analgesics and antibacterial drugs were also found to have a high incidence of discrepancies following the administration of cardiovascular agents. Pain management is the cornerstone of ERAS programs, and antibiotics are essential both for infection prophylaxis/treatment; these related medications are widely used perioperatively. Contraindications were the most common type of discrepancy, probably because with multiple chronic diseases, older people are at a greater risk for drug interactions and contraindications brought by polypharmacy [19]. Therefore, cardiovascular agents, analgesics and antibacterial drugs carry the highest risk of discrepancies, and contraindications with their high prevalence require special attention from caregivers.

Using the Bayliff tool, 71.9% of unintentional medication discrepancies were identified as having potential clinical impacts that led to further treatment or lengthened hospital stays, and 10.3% were related to life-threatening hazards. The risk found was somewhat different from the study of Nashville, where 75.2% were categorized as significant in severity, 22.9% were serious, and 1.8% were life-threatening [20]. The differences could be explained by the fact that in our study, MR was implemented in an ERAS population with older age who went through a major surgery. These results show that MR has a promising effect in the prevention of errors that may contribute to devastating harm in older patients undergoing orthopedic joint surgery.

In the second stage of the study, MR reduced the average length of hospital stay and unplanned visits after discharge in the group of patients diagnosed with PJI. Previous studies [21, 22] have also shown that MR contributes to a reduction in unplanned emergency department visits and readmission to the hospital within 30 days for all hospitalized adults. However, no clear evidence was found previously in favor of MR in reducing length of stay reported by a review [23]. One possible reason for this outcome is that our study included PJI patients scheduled for two-stage revision. PJI represents one of the most devastating complications in joint arthroplasty and is associated with a prolonged hospital stay with debridement surgery and long-term intravenous antibiotic use. Through pharmacist assistance in antibiotic management, optimizing medication regimens, e.g., selecting medication, dosage and frequency based on pathogens and patient’s hepatic and renal function, as well as serum drug concentration monitoring, a better pharmacotherapy outcome was achieved. This may probably have contributed to the reduction in length of hospital stay.

Similarly, MR shortened the time between the first admission and the reintroduction of joint replacement and saved the care cost. Most PJI patients required long-term antibiotic therapy and thus were unable to complete the whole treatment process in our hospital [24]. In China, tertiary medical treatment and two-way referral policy require the transference of patients from higher grade hospitals to lower grade hospitals once the patient’s status is stabilized. However, the transfer itself carries the risk of deviating from or discontinuing the original effective pharmaceutical treatment plan for two main reasons. First, evaluation and treatment could be different from doctors of different facilities, including types of antibiotics, frequency and dose. Second, pharmacy drug lists may differ greatly at different facilities. By obtaining drug information from the post-acute care facilities and MR before discharge, adherence to the original pharmacotherapeutic plan was easier. This may contribute to reducing the time between the first admission and the reimplantation of a new prosthesis. MR also improved patient satisfaction with medical services, especially in terms of pain, nausea and vomiting control, which in turn improved the outcome of the ERAS program.

The major limitation of this study was that not all orthopedic joint surgeries were randomized for comparison. As the controlled group carried a potential risk of medication errors, the comparative study was narrowed down to patients diagnosed with periprosthetic joint infection (PJI) and scheduled planned for two-stage revision. Thus, the conclusion about the improved outcome can only be narrowed to PJI patients. Additionally, the studied groups did not use a blind method because the patients and caregivers in the surgery unit obviously knew about the intervention of pharmacists. However, this may add bias to the results, especially with patients reporting symptoms or their satisfaction with the surgery.

In conclusion, pharmacist-led MR prevented medication discrepancies in orthopedic ERAS programs and achieved a better surgical outcome as well as patient satisfaction in patients with PJI. Although the findings should be interpreted in the context of the study’s limitations, this study provides information for hospitals and surgical practices interested in implementing and evaluating enhanced recovery programs and minimizing misuse or overuse of medications, improving outcomes and decreasing costs.

## Data Availability

The datasets used and/or analysed during the current study are available from the corresponding author on reasonable request.

## Abbreviations

PJI: Periprosthetic joint infection
ERAS: Enhanced recovery after surgery
ID: Intentional discrepancy
UD: Unintentional discrepancy
SD: Standard deviation
MR: Medication reconciliation
ADRs: Adverse drug reactions
